# Catch damage in broiler chickens: a systematic review of injury assessment methods and their limitations

**DOI:** 10.1016/j.psj.2026.107825

**Published:** 2026-09-21

**Authors:** Patryk Sztandarski, Joanna Marchewka, Aneta Jaszczyk, Magdalena Solka, Jarosław O. Horbańczuk, Anja B. Riber, Frank A.M. Tuyttens, T. Bas Rodenburg

**Affiliations:** a Institute of Genetics and Animal Biotechnology, Polish Academy of Sciences, Jastrzebiec, Magdalenka, 05-552, Poland; b Department of Animal and Veterinary Sciences, Aarhus University, Tjele, DK-8830, Denmark; c Animal Sciences Unit, Flanders Research Institute for Agriculture, Fisheries and Food (ILVO), Merelbeke-Melle, Belgium; d Department of Veterinary and Biosciences, Faculty of Veterinary Medicine, Ghent University, Merelbeke-Melle, Belgium; e Department of Population Health Sciences, Faculty of Veterinary Medicine, Utrecht University, Utrecht, 3584 CL, the Netherlands; f Adaptation Physiology Group, Department of Animal Sciences, Wageningen University & Research, P.O. Box 338, 6700 AH Wageningen, the Netherlands

**Keywords:** Catch damage, Broiler, Welfare, Injury assessment, Bruise

## Abstract

Catch damage in broiler chickens is widely used as an indicator of preslaughter welfare and carcass quality, yet its assessment methods vary considerably across the scientific literature. The objective of this review was to systematically evaluate how catch damage is defined, measured, and attributed in studies involving broiler chickens, with particular attention to methodological consistency and interpretational limitations. A structured literature search identified peer -reviewed studies addressing physical injuries associated with catching and preslaughter handling. Extracted information included injury definitions, assessment stage, scoring systems, sampling strategies, and approaches used to determine injury origin. The review revealed that catch damage is assessed using diverse and often non-comparable frameworks, including on-farm examinations, lairage inspections, postmortem slaughter line scoring, processing record analysis, automated image-based detection, and physiological or biochemical indicators. Catch damage definitions differed in anatomical scope, lesion size thresholds, counting rules, and aggregation level, while many studies relied on postmortem proxy measures such as bruising prevalence, fractures, condemnations, or dead-on-arrival (DOA) rates. These outcomes frequently represent cumulative trauma from multiple preslaughter stages rather than catching specific injury. Limited differentiation between recent and old injuries further constrains stage-specific attribution. Inconsistent reporting of observer training, reliability, and sampling design was also common. Collectively, these methodological differences contribute to measurement bias and restrict cross-study comparability, causal inference, and the development of evidence-based welfare interventions. Catch damage therefore not only represents a biological outcome, but also a measurement construct influenced by assessment context and protocol design. Greater harmonization of injury definitions, injury age classification, and standardized scoring procedures is needed to improve the reliability and practical applicability of catch damage severity as a welfare and quality indicator in broiler production.

## Introduction

The welfare of poultry, particularly broiler chickens, during catching and preslaughter handling has become an increasingly important focus in both scientific research and commercial poultry production (Lambrecht et al., 2020). Catch damage, defined as physical injury sustained during catching, crating and related handling procedures, represents a critical welfare concern. It is also associated with economic losses due to carcass downgrading, condemnations, and reduced product quality (De Lima et al., 2019; Gouveia et al., 2009; Jacobs and Tuyttens, 2020; Wilcox et al., 2024). As modern poultry systems continue to intensify, the ability to not only reduce catch-related injuries, but also to measure them accurately has become essential for protecting animal welfare and ensuring efficient production and maintaining carcass quality. An increasing number of publications analyze catch damage as an outcome of preslaughter catching and crating. Numerous studies have investigated associations between catching practices, stocking density, body weight, transport conditions, and injury prevalence (Jacobs et al., 2017; Smith and Pierdon, 2025; Van Emous et al., 2024). These studies consistently demonstrate that catching is a high-risk phase for physical trauma, including bruising, wing and leg injuries, dislocations, and fractures. Such injuries not only compromise immediate welfare but also increase stress and susceptibility to further injury during transport, which negatively affects meat quality at slaughter (Chauvin et al., 2011; Gou et al., 2021; Schilling et al., 2008). Catch damage therefore functions simultaneously as a welfare indicator, a production outcome, and a quality-control parameter.

However, beyond documenting prevalence and associated risk factors, the literature highlights a more fundamental issue. An important methodological concern is that catch damage may not represent a single, clearly defined outcome, as its assessment can vary according to injury definitions, detection methods, and the stage at which injuries are evaluated. Injuries observed at slaughter may reflect trauma occurring during several sequential preslaughter stages, potentially complicating the attribution of damage to catching itself. Differences in how injuries are defined, detected, and attributed may therefore influence the interpretation and comparability of catch damage data across studies. Systematically evaluating these methodological differences is necessary to determine the extent to which they affect the assessment of catching-related injuries.

A major limitation is that catch damage is rarely measured directly during the catching process. Instead, most studies infer catching-related injury from postmortem assessments at the slaughterhouse, using bruising, fractures, condemnations, or dead-on-arrival (DOA) rates as proxy indicators (Averós et al., 2020; Saraiva et al., 2020). Although this approach is operationally convenient and aligns with commercial inspection systems, it inherently conflates injuries originating from catching with those incurred during subsequent stages (crating, loading, transport, unloading, lairage, shackling, and stunning) (Buzdugan et al., 2020; Petracci et al., 2010). Multiple authors acknowledge that bruises and fractures detected after plucking cannot be reliably attributed to catching alone, thereby weakening causal inference about the effects of specific preslaughter handling conditions (Jacobs et al., 2017; Nijdam et al., 2006; Pitcovski et al., 1997). Consequently, many commonly used indicators function as stage-aggregated outcomes rather than stage-specific measures.

Another critical methodological gap concerns the lack of timing differentiation between fresh and old injuries. From an industry perspective, distinguishing fresh injuries attributable to recent handling events from older injuries originating during rearing is essential for accurate welfare assessment, corrective management, and accountability within the production chain. However, many studies do not report injury age, and several explicitly state that no distinction was made between fresh and old (Hassan and Lashin, 2017; Jacobs et al., 2017; Malpass et al., 2010). This limitation reduces the ability to link observed injury to specific handling stages and undermines the usefulness of injury data for targeted welfare interventions (Cockram et al., 2018; Hamdy et al., 1961a; Malpass et al., 2010). The absence of temporal resolution therefore represents a key barrier to valid injury attribution.

Potential sources of methodological variation in catch damage assessment include differences in injury definitions, scoring thresholds, and anatomical regions assessed. Catch damage outcomes vary according to how injuries are defined, quantified, and aggregated, including differences in lesion size criteria, anatomical coverage, and reporting level (e.g., carcass, anatomical region, flock, or batch). Assessment approaches may also differ in the types of injuries considered and the degree of standardization and documentation of procedures. Some studies apply established welfare assessment frameworks, such as Welfare Quality (2009) or EFSA AHAW Panel, 2023, whereas others rely on ad hoc scoring systems or company specific quality assurance procedures without clear methodological justification (Delanglez et al., 2025; Langkabel et al., 2015; Pilecco et al., 2013). These variations introduce measurement bias and limit both reproducibility and cross-study comparability.

Addressing these issues therefore requires a systematic evaluation not only of catch damage outcomes, but of the methods used to generate those data, with particular attention to how measurement choices influence injury detection, attribution, and interpretation.

The aim of this review is to evaluate the methods used to assess catch damage in broiler chickens, with a particular focus on how catch damage is defined, measured, and attributed within the literature. Specifically, this review compares on farm, postmortem, and combined approaches used to assess catch damage. It also identifies commonly used scoring protocols, evaluation methods and discusses their limitations. Finally, the review highlights key methodological gaps and inconsistencies that restrict accurate identification of catching-related injuries, including the timing of catch damage occurrence.

## Materials and methods

### Literature search strategy

A comprehensive systematic literature search was conducted to identify relevant studies that discuss the incidence, underlying factors, and assessment methods of catch damage in broiler chickens. The databases searched included Scopus, PubMed, and Web of Science, using a combination of keywords and Boolean operators to capture the broadest relevant literature. The search terms used were as follows: ("catch damage" OR "handling injury" OR "transport injury" OR “catch lesions”) AND ("broiler chicken*" OR "broilers") AND ("welfare indicators" OR "animal welfare" OR "poultry welfare") AND ("underlying factors" OR "risk factors" OR "causes") AND ("types of damage" OR "injury types" OR "damage assessment" OR “lesion”) AND ("assessment methods" OR "evaluation methods" OR "damage evaluation" OR “damage protocol evaluation” OR “lesion identification”). The database searches were conducted on January 7, 2026. The search was limited to articles published in English from January 1997 to December 2025. An additional Scopus search without date restrictions was conducted to capture earlier methodological research on bruise aging. This search retrieved 2 publications from 1961.

Both records were evaluated using the same screening and eligibility criteria as those retrieved during the primary search and were included in the final set of 26 studies. The search and study selection process was reported in accordance with the PRISMA 2020 recommendations (Page et al., 2021).

### Selection criteria and screening procedure

Inclusion criteria were established to identify peer-reviewed studies that specifically addressed physical injuries in broiler chickens in relation to catching and closely associated preslaughter handling processes, including studies describing assessment methods applied at the farm and slaughterhouse levels. Although individual injuries such as bruises, hematomas, fractures, dislocations, and wing injuries are widely reported in broilers, studies were included only when these outcomes were explicitly linked to catching or related handling stages and provided sufficient methodological information to evaluate how catch damage was defined, measured, or attributed. Studies that did not directly assess physical injuries, or that addressed injuries without a clear relationship to catching or associated preslaughter handling, were excluded.

Records retrieved from all databases were combined. Duplicate records were identified by matching DOI, PMID, and normalized article titles and were removed before screening. Titles and abstracts were screened against the predefined eligibility criteria, followed by full-text assessment of potentially eligible reports. Screening and eligibility assessment were performed independently by 2 reviewers. Any disagreements were resolved through discussion or, when necessary, consultation with a 3rd reviewer. The literature search and study selection process is summarized in the PRISMA 2020 flow diagram presented in Fig. 1.

**Fig. 1:**
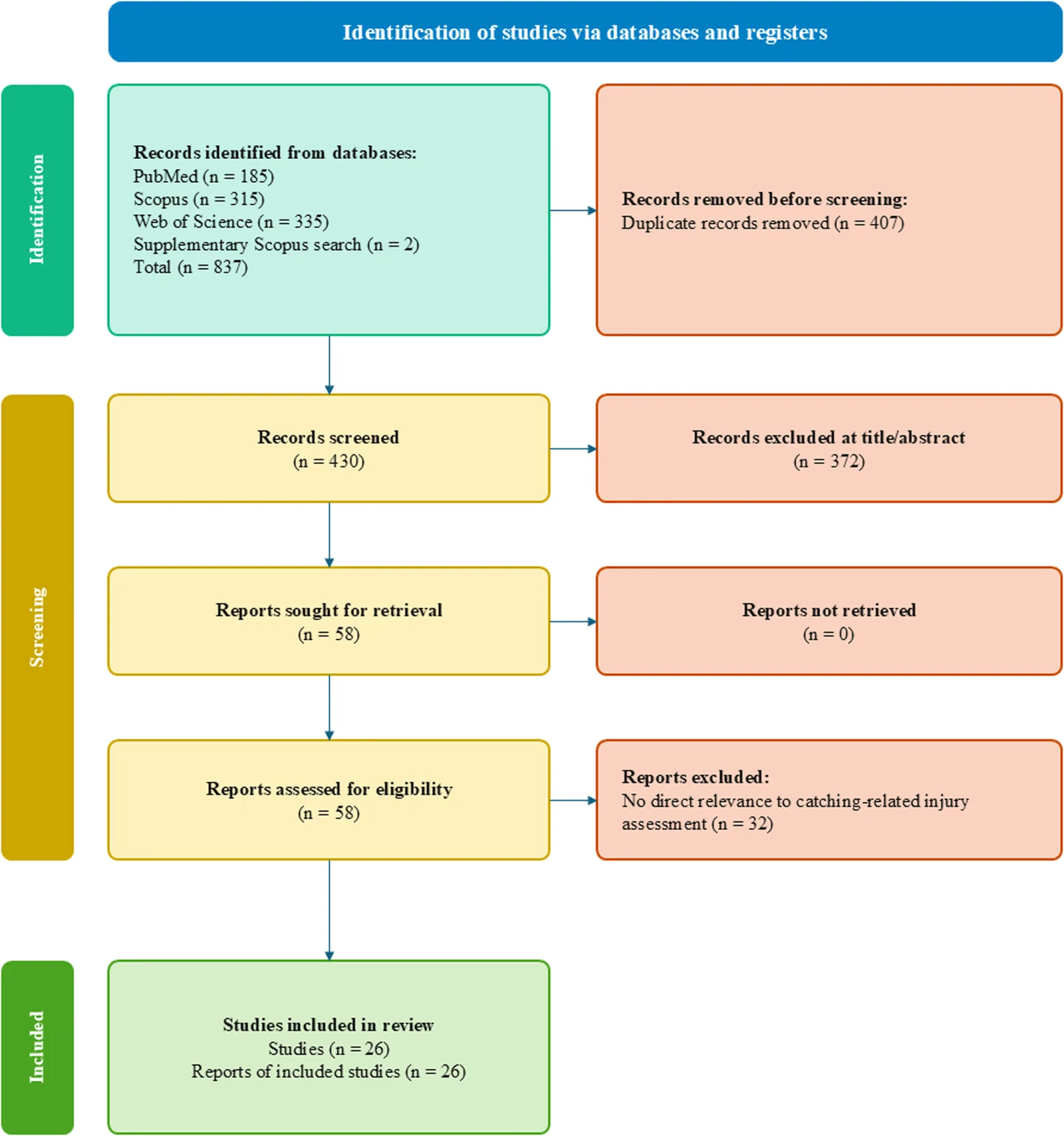
PRISMA flow diagram of the literature search and study selection process. The database searches, including a supplementary Scopus search without a publication-year restriction, identified 837 records. After removal of 407 duplicate records, 430 records were screened based on their titles and abstracts, of which 372 were excluded. All 58 reports sought for retrieval were obtained and assessed for eligibility. Following full-text assessment, 32 reports were excluded because they were not directly relevant to catching-related physical injury assessment. Ultimately, 26 studies, represented by 26 reports, were included in the review.

### Methodological quality assessment

A formal risk of bias or methodological quality assessment tool was not applied because the objective of the review was to compare and critically evaluate methods used to define, measure, and attribute injuries associated with catching rather than to estimate a pooled intervention effect. The included studies were highly heterogeneous in terms of study design, assessment stage, injury definitions, sampling procedures, and reporting units, which made it inappropriate to evaluate all studies using the same methodological quality framework. The extracted data were qualitatively synthesized to identify common themes and patterns regarding the underlying causes and types of catch damage, as well as to evaluate the different assessment methods used across studies. Meta-analytic methods were not used because most of the extracted data were qualitative and only a limited number of articles were available within each thematic group.

## Results

### Articles overview

The database searches identified 837 records, including 2 records retrieved through a supplementary Scopus search without a publication-year restriction. After removal of 407 duplicate records, 430 records were screened based on their titles and abstracts, of which 372 were excluded. All 58 reports sought for retrieval were obtained and assessed for eligibility. Of these, 32 were excluded because they were not directly relevant to catching-related physical injury assessment, resulting in 26 studies being included in the final review database (Fig. 1). 2 studies on bruise aging were published in 1961 (Hamdy et al., 1961a, 1961b), whereas the remaining 24 studies were published between 1997 and 2025. The distribution by publication period was as follows: 1961 (n = 2), 1997 to 2004 (n = 4), 2005 to 2014 (n = 5), and 2015 to 2025 (n = 15) (Fig. 2A). The studies were published across 12 different peer reviewed journals (Fig. 2B), with *Poultry Science* representing the largest share (n = 13). Additional studies were published in the *Journal of Applied Poultry Research* (n = 2). The remaining studies were distributed across several other journals covering poultry science, veterinary science, animal welfare, and food science. By study focus, the reviewed literature (Fig. 2C) included investigations assessing carcass injuries or bruising at slaughter (n = 8), comparisons of manual and mechanical catching methods (n = 5), analyses of preslaughter handling risk factors (n = 5), studies addressing stage-specific attribution of injuries (n = 4), and investigations examining physiological responses associated with catching and handling practices (n = 4). The methodological approaches used in these studies are summarized in Table 1, and strategies applied to estimate the timing and attribution of injuries are presented in Table 2.

**Fig. 2:**
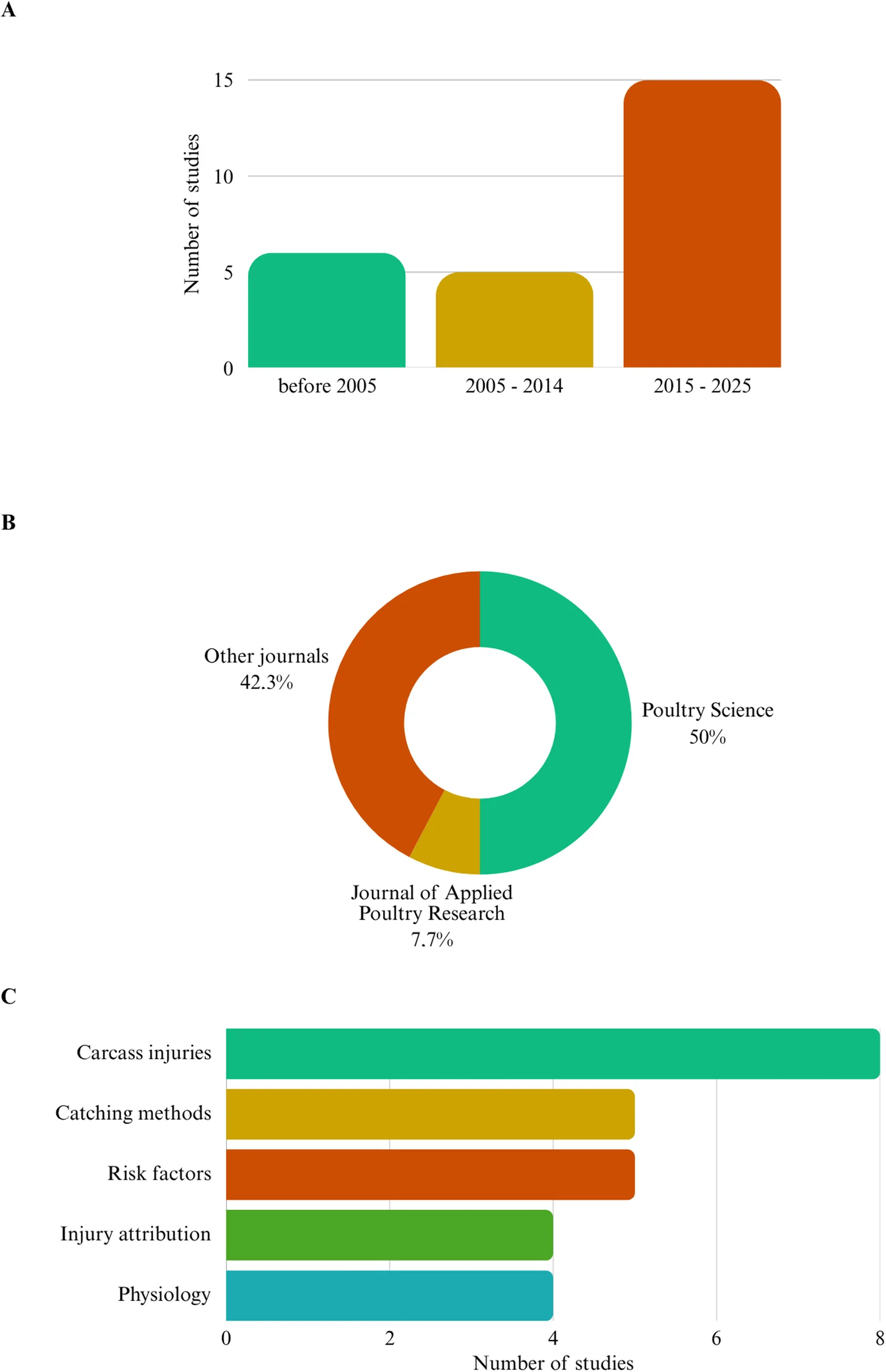
**Overview of the studies included in the review**. (A) Distribution of publication years of the studies included in the analysis.(B) Journal distribution of the included publications, highlighting the proportion of studies published in Poultry Science, the Journal of Applied Poultry Research, and other journals.(C) Main research focus of the included studies, categorized into: carcass injury, catching methods, risk factors, injury attribution, physiology.

**Table 1:** Methodological approaches used to assess catch damage in broiler chickens.

Assessment Approach	Measurement Stage	Method / Tools Used	Injury Definitions & Thresholds	Scoring System	Key References
On-farm post catching assessment	Immediately before and / or after catching and loading on-farm	Live chicken clinical examination combining visual inspection and palpation	Minor injuries defined as hematomas ≥ 0.5 cm on wings, body or legs; severe injuries ≥ 0.5 cm with fractures or dislocations detected by palpation	Binary presence, absence scoring per anatomical location; summarized at flock level	Mönch et al., 2020; Wessel et al., 2022
Lairage examinations for injury assessment	Arrival at slaughterhouse before stunning	Manual examination of chickens removed from transport modules	Wing fractures defined as open, closed fractures, epiphyseal plate detachment with bleeding, or wings hanging vertically downward; dislocations included	Binary presence, absence scoring per chicken; prevalence calculated at flock	Kittelsen et al., 2015a, 2018
Postmortem slaughter line scoring systems assessment	After plucking in evisceration or inspection area	Visual inspection of carcasses on slaughter line; observers assigned to specific body regions	Fresh bruises ≥1 cm with red discoloration and no yellow, green tones; fractures defined by bone protrusion or abnormal structure accompanied by hemorrhage	Binary presence, absence or categorical scoring; injuries counted per carcass or per anatomical region	Delanglez et al., 2025; Saraiva et al., 2020
		Visual scoring according to legislative or industry standards (e.g., Dutch PVE system)	Bruises defined as blood discoloration > 1 cm² for breast, leg and > 2 cm² for wings	Binary presence, absence scoring per anatomical region; wings and legs counted separately	Nijdam et al., 2005; PVE (2001)
Slaughterhouse record-based monitoring assessment	Slaughterhouse inspections systems	Data derived from slaughterhouse quality control systems such as the IKB Kip certification scheme	Defined thresholds (e.g., wing hematoma > 2 cm², leg hematoma > 1 cm², breast bleeding > 1 cm²)	Prevalence (%) of chickens affected per transport vehicle or batch	van Emous et al., 2024; IKB Kip (2017)
	Batch or truckload level processing records	Analysis of slaughterhouse inspection data across large datasets	Bruising defined using region specific thresholds (e.g., > 2 cm² wings or > 1/3 of breast/leg area)	Carcass level aggregation rule; prevalence reported per batch or truckload	Pirompud et al., 2023
	Unloading or processing stage	Analysis of DOA records with transport and environmental variables	No direct injury definition; mortality used as proxy for preslaughter welfare compromise	Percentage DOA per trailer load	Caffrey et al., 2017
Automated image-based detection systems assessment	Slaughter line after plucking	Camera-based machine vision systems detecting discoloration and wing deformation	Hemorrhage detection using RGB color analysis; fractures identified through abnormal wing angle relative to expected axis	Binary lesion detection combined with ordinal severity categories describing hemorrhage extent (< 30%, 30 to 60%, > 60% of the affected anatomical region) and hemorrhage intensity (mild medium or severe) based on RGB color analysis.	Langkabel et al., 2015
Physiological and biochemical indicators assessment	Sequential preslaughter stages (catching, crating, transport)	Blood sampling and physiological monitoring (corticosterone, glucose, lactate, CK, electrolytes, H/L ratio)	No direct injury definition; CK interpreted as indicator of muscle strain or tissue damage	Sequential preslaughter handling stages (e.g., before catching, loading, after catching or crating, loading, and after transport upon arrival at the slaughterhouse).	Nijdam et al., 2005; Vošmerová et al., 2010; Yalçin et al., 2004

DOA (dead-on-arrival), RGB (Red, Green, Blue), H/L (heterophil to lymphocyte ratio), CK (creatine kinase).

**Table 2:** Methodological approaches used to estimate the timing and attribution of catch damage in broiler chickens.

Assessment Approach	Measurement Stage	Method / Tools Used to Estimate Injury Age	Injury Definitions & Thresholds	Scoring System	Key References	
On-farm post catching assessment	Immediately after catching	Visual inspection and palpation by observer, color differentiation of hematomas	Hematomas defined by color (red, dark red, green)	Binary presence, absence scoring: injuries recorded per chicken and anatomical region (wing, body, legs). Hematoma color used as indicator: red, dark red = fresh, greenish = older injury.	Wessel et al., 2022	
Color-based bruise ageing during slaughter monitoring assessment	After plucking in evisceration or inspection area	Visual inspection of bruise color, morphology, and anatomical distribution by trained slaughterhouse observers	Injuries recorded as bruising of wings, legs, or breast, hip dislocation, or shoulder damage	Injuries classified into 3 origin categories: (1) Old, rearing injuries – green, yellow bruises or lesions with healing appearance; (2) Recent handling, transport injuries – red or dark red bruises with hemorrhage; (3) Processing injuries – damage without hemorrhage or typical of equipment. Data recorded as % of chickens affected per trailer load	Cockram et al., 2020	
			Fresh bruises ≥ 1 cm diameter with red coloration and no yellow, green discoloration; fresh fractures defined by bone break with surrounding redness or bluish hemorrhage	Binary presence, absence scoring: wings, legs, breast, wing tip: (1) bruise + fracture counted as fracture; (2) multiple injuries of same type per region qualified to one injury; (3) fractures without hemorrhage classified as processing damage and excluded	Delanglez et al., 2025	
		Visual inspection of bruise color guided by descriptions derived from experimental studies	Bruises identified as injury caused by hemorrhage; color categories linked to approximate age (e.g., bright red vs dark vs green, yellow)	Bruises categorized according to estimated time of occurrence: (1) Recent preslaughter injuries (red, dark red); (2) Older injuries likely from rearing (green, yellow). Only injuries attributed to preslaughter period included in catch damage analysis	Northcutt et al., 2000; Saraiva et al., 2020	
Experimental bruise age validation framework	Controlled experimental bruising followed by slaughter line evaluation	Objective color measurement using colorimeter (CIE L*a*b*), and histological examination of muscle tissue	Bruises experimentally induced at known times prior to slaughter; no strict size threshold but standardized injury induction	Bruise age classified using visual color progression and CIE color differences (ΔL, Δa, Δb). 0 to 1 h: bright red bruise with localized hemorrhage. 6 h: bruise becomes darkest (maximum ΔL difference), corresponding with peak tissue edema. 12 h: dark red to purple coloration, reduced redness values. 24 h: green discoloration due to hemoglobin degradation (biliverdin formation).	Northcutt et al., 2000	
	Controlled experimental bruising	Visual observation of bruise color progression over time	Bruises experimentally induced to observe tissue response	Descriptive ageing framework: 0 to 2 min: no visible color; minutes to hours: red; ∼12 h: dark red, purple; ∼24 h: green discoloration due to hemoglobin degradation. Used as conceptual basis for later slaughterhouse scoring systems	Hamdy et al., 1961a, 1961b	

### Definitions of catch damage

Across studies, catch damage was operationalized using several types of definitions, including postmortem bruising assessment, musculoskeletal injuries assessment, slaughterhouse indicators, and physiological or behavioral responses associated with preslaughter handling. The main categories of catch damage definitions identified in the literature are summarized in Fig. 3.

**Fig. 3:**
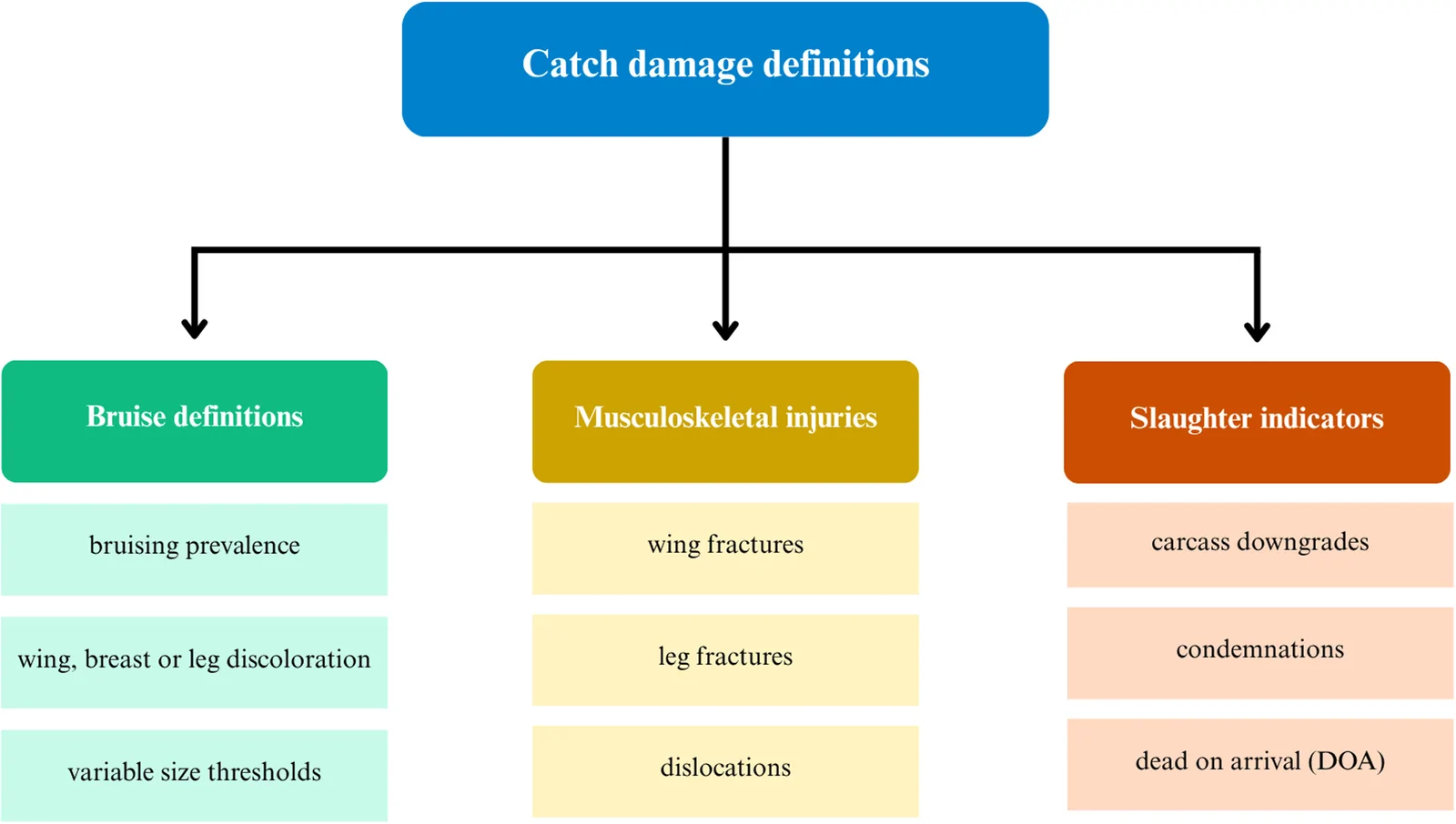
Conceptual classification of catch damage definitions used in the literature. The figure illustrates the main conceptual approaches used to define catch damage in broiler chickens. In the literature, catch damage is commonly described based on bruising-related outcomes detected after plucking, musculoskeletal injuries such as fractures or dislocations, and indirect slaughterhouse indicators including carcass downgrades, condemnations, and dead-on-arrival (DOA) birds. These definitions differ in injury type, assessment stage, and diagnostic criteria.

Postmortem bruising assessment, typically done after plucking, was the most frequently reported injury outcome (Pirompud et al., 2023; Saraiva et al., 2020). Operational definitions varied in lesion thresholds and reporting units. Nijdam et al. (2005) applied thresholds of > 1 cm² for breast and leg bruises and > 2 cm² for wing bruises, whereas Delanglez et al. (2025) included fresh bruises ≥ 1 cm showing red discoloration. Other studies recorded injuries as presence-based outcomes without quantitative size thresholds (Hassan and Lashin, 2017).

Musculoskeletal injuries, including fractures and dislocations, were assessed after loading or during lairage (Kittelsen et al., 2015a,; 2018; Wessel et al., 2022), whereas large-scale studies used aggregated slaughterhouse-derived outcomes aggregated at the flock, batch, transport-load, or vehicle level (Nijdam et al., 2006; Pirompud et al., 2023; van Emous et al., 2024). Only a minority of studies differentiated injuries according to their presumed timing or stage of occurrence, using comparisons between successive stages, lesion color and morphology, anatomical location, or acute and chronic injury classifications (Cockram et al., 2020; Delanglez et al., 2025; Jacobs et al., 2017; Malpass et al., 2010). Behavioral and physiological variables were reported as associated preslaughter outcomes rather than structural injury measures (Vošmerová et al., 2010; Wessel et al., 2022; Yalçin et al., 2004).

### Methodological approaches to catch damage assessment

Among the 26 included studies, the primary research focus was carcass injury or bruising in 8 studies (30.8%), comparisons of catching methods in 5 studies (19.2%), preslaughter handling risk factors in 5 studies (19.2%), stage-specific injury attribution in 4 studies (15.4%), and physiological responses associated with catching and handling in 4 studies (15.4%). These mutually exclusive categories describe the principal focus of each study; however, individual studies frequently combined several assessment stages and outcome types. Across the reviewed literature, 6 recurring methodological frameworks were identified: on-farm examinations, lairage examinations, slaughter line scoring, slaughterhouse monitoring, automated detection systems, physiological and biochemical indicators (Fig. 4 and Table 1).

**Fig. 4:**
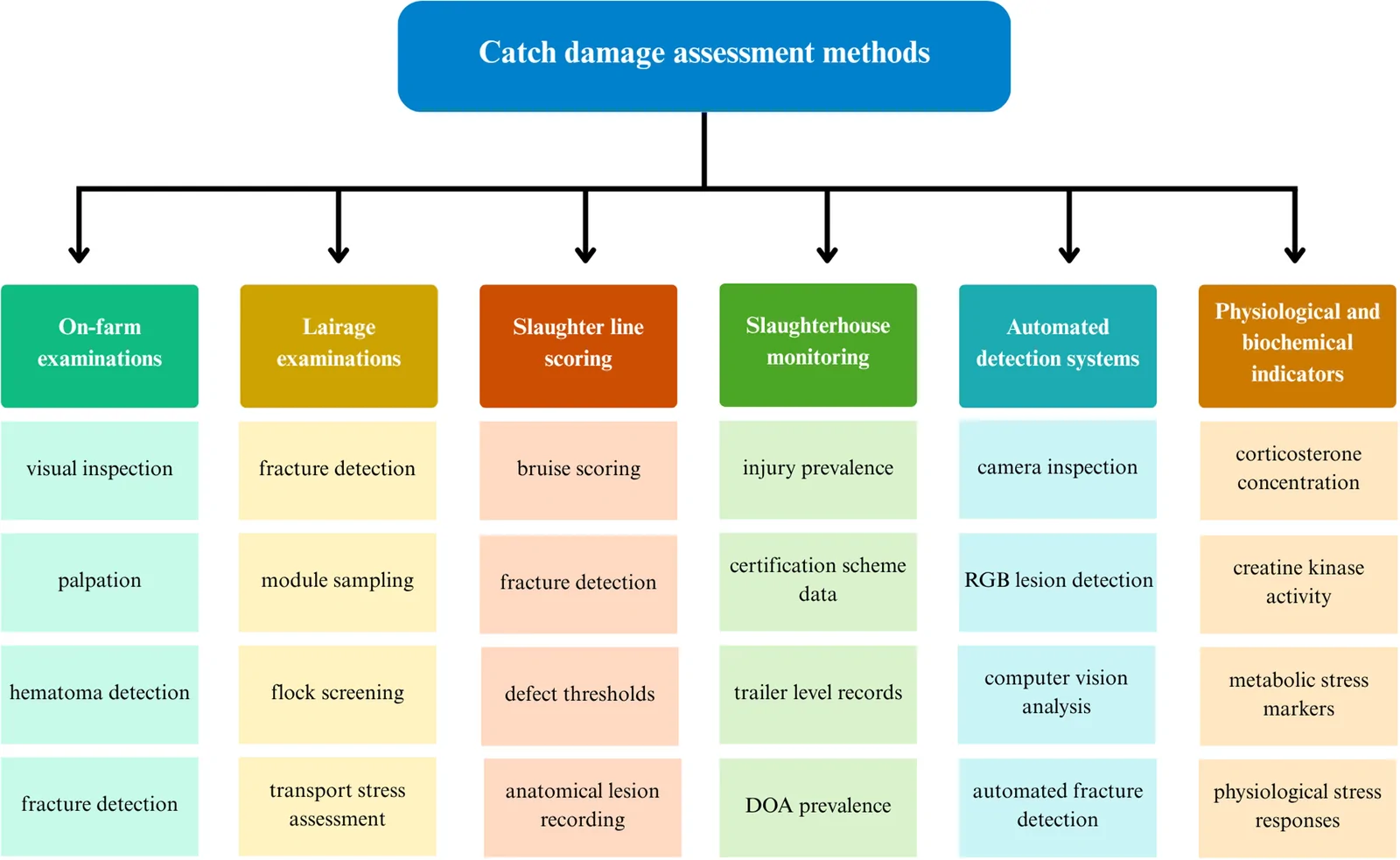
Methodological approaches used to assess catch damage in broiler chickens. The figure summarizes the main methodological frameworks used in the literature to evaluate catching-related injuries. These include on-farm examinations of live birds, lairage based injury assessment, postmortem slaughter line scoring systems, slaughterhouse monitoring, automated image detection systems, and physiological or biochemical indicators of preslaughter stress. Each approach differs in the stage of assessment, type of measured outcomes, and level of methodological standardization.

### On-farm and immediate post catching assessment

Examinations performed on-farms focused on live broilers assessed immediately after catching and loading. These protocols generally combined visual inspection for hematomas with palpation for fractures, dislocations, or other severe wing injuries. Outcomes were usually recorded as binary variables according to anatomical region and subsequently summarized at flock level. Mönch et al. (2020) and Wessel et al. (2022) applied related protocols using predefined injury criteria. Wessel et al. (2022) additionally incorporated structured sampling from different barn sections and behavioral observations during catching.

### Lairage examinations for injury assessment

In some studies upon arrival at the slaughterhouse, selected broilers were removed from transport modules and examined before stunning. The principal outcomes were wing and leg fractures, dislocations, and DOA. Kittelsen et al. (2015a) used explicit examination criteria for wing fractures, including open or closed fractures, epiphyseal plate detachment with visible bleeding, dislocation, and abnormal wing position. Kittelsen et al. (2018) applied related criteria while tracking marked transport modules, allowing injuries and mortality to be related to the catching method and transport unit. Results were expressed as the prevalence of affected broilers at flock or module level.

### Postmortem slaughter line scoring systems assessment

In other studies following slaughter and plucking, carcasses were examined for bruises, fractures, dislocations, and other visible injuries. Injuries were classified according to anatomical location and reported as binary or categorical outcomes per carcass, body region, or flock. The degree of standardization differed considerably among protocols. Some studies applied predefined size, color, or morphological criteria, whereas others recorded the presence of visible damage without quantitative thresholds. Specific definitions of bruising also varied between studies. Nijdam et al. (2005) defined bruises as blood related discoloration larger than 1 cm² on the breast or legs and larger than 2 cm² on the wings. Delanglez et al. (2025) included fresh bruises measuring at least 1 cm and showing red discoloration, whereas yellow or green lesions and fractures without surrounding hemorrhage were excluded. Saraiva et al. (2020) classified bruises according to anatomical region and apparent age, whereas Hassan and Lashin (2017) recorded visible bruises and fractures without quantitative size criteria. A different postmortem approach was applied by Kittelsen et al. (2015b), who conducted systematic necropsies of broilers classified as dead on farm or DOA and recorded traumatic lesions using predefined diagnostic categories.

### Slaughterhouse record-based monitoring assessment

Rather than examining every carcass individually, several studies analyzed injury and mortality indicators at the transport load, truckload, or flock level. The main outcomes included bruising prevalence, carcass condemnations, and DOA. These studies differed in lesion thresholds and aggregation rules. Pirompud et al. (2023) used body area-specific criteria but classified a carcass as bruised when a qualifying lesion occurred in any assessed anatomical region. Van Emous et al. (2024) reported the percentage of affected broilers per transport vehicle using standardized thresholds for wing hematomas, leg hematomas, and breast bleeding. In contrast, Caffrey et al. (2017) used DOA prevalence per trailer load as the principal preslaughter outcome and related it to transport, environmental, and flock-level variables.

### Automated image-based detection systems assessment

Only 1 of the 26 included studies (3.8%) used automated image analysis to assess injuries. Langkabel et al. (2015) applied a camera system positioned after plucking to detect hemorrhages and abnormal wing position. Lesions were recorded as binary outcomes and further classified according to their extent and intensity. Hemorrhages were identified using RGB color analysis, whereas wing fractures were detected based on deviations in wing position. The automated classifications were calibrated and validated against independent human assessor visual assessments.

### Physiological and biochemical indicators assessment

Physiological and biochemical measurements constituted the primary focus of 4 studies (15.4%). The variables used to evaluate responses to catching and handling included corticosterone, body temperature, blood gases, electrolytes, glucose, lactate, creatine kinase (CK), and the heterophil to lymphocyte ratio (H/L) (Jacobs et al., 2017; Nijdam et al., 2005; Vošmerová et al., 2010; Yalçin et al., 2004). Measurements were collected at defined stages before or after catching, crating, loading, transport, and arrival at the slaughterhouse. Some protocols combined physiological measurements with direct carcass injury assessment, whereas others used physiological variables as associated preslaughter outcomes without recording structural injuries.

### Timing and attribution of catch damage injuries

6 studies specifically contributed to the assessment of injury timing or origin (Hamdy et al., 1961a, 1961b; Kittelsen et al., 2015a; Pitcovski et al., 1997, 2015b; Cockram et al., 2020). Across these and other included studies, 3 general approaches were used to estimate when and where injuries occurred: comparisons between successive production stages, macroscopic assessment of lesion color and morphology, and experimentally validated criteria for estimating bruise age (Table 2). Comparisons conducted before and after specific handling or processing stages were used to distinguish injuries associated with catching, transport, lairage, or slaughterhouse processing (Cockram et al., 2020; Kittelsen et al., 2015a; Malpass et al., 2010; Pitcovski et al., 1997). Other studies classified red or dark red lesions as recent and green or yellow lesions as older injuries (Delanglez et al., 2025; Saraiva et al., 2020; Wessel et al., 2022). The interpretation of these macroscopic characteristics was based partly on controlled studies of bruise aging that evaluated colorimetric and histological changes in lesion appearance over time (Hamdy et al., 1961a, 1961b; Northcutt et al., 2000).

## Discussion

The present review examined the methodologies used to assess catch damage in broiler chickens, with emphasis on how injuries are defined, detected, and quantified. The primary focus was on mapping the assessment frameworks applied in practical catching contexts, including on-farm examinations immediately before or after loading, lairage inspections, slaughter line injury scoring, slaughterhouse record-based monitoring, and combined physiological approaches. In doing so, the review targeted empirical studies describing catching-related conditions. The results show considerable methodological variation in the assessment of catch damage, including differences in assessment stage, injury thresholds, counting rules, and attribution approaches. Such differences may influence the reported catch damage outcomes and their interpretation, supporting the view that catch damage assessment is shaped not only by the occurrence and characteristics of injuries, but also by the methodological framework used to detect, quantify, and attribute them.

Within the current literature, catch damage represents a composite and context dependent term referring to several types of outcomes: acute mechanical trauma (bruises, fractures, dislocations), cumulative preslaughter injuries detected at slaughter, and indirect quality indicators such as condemnations and DOA. These categories differ in pathogenesis, timing, and detection sensitivity. The absence of harmonized catch damage definitions, standardized anatomical scoring systems, and stage-specific attribution therefore limits comparability across studies and obscures the true contribution of catching itself to observed welfare and quality outcomes. This lack of clear definitions is not only a matter of terminology, but also strongly affects how causes are identified, how interventions are designed, and how evidence-based catching practices are developed.

The most frequently used definition of catch damage was based on postmortem bruising prevalence. Despite its widespread use, assessment protocols differed in lesion size thresholds, anatomical coverage, reporting units, and rules for counting multiple injuries on the same broiler (Delanglez et al., 2025; Malpass et al., 2010; Pirompud et al., 2023; Saraiva et al., 2020). Consequently, reported bruising prevalence depends on the applied scoring rules, which limits direct comparisons between studies.

A second definitional approach interprets catch damage in structural terms, focusing on skeletal and joint trauma such as wing fractures, dislocations. These injuries are interpreted as direct consequences of mechanical strain during handling and are sometimes detected immediately after loading or during lairage examinations (Kittelsen et al., 2015a; Langkabel et al., 2015; Wessel et al., 2022). This framework shifts attention from surface discoloration to musculoskeletal integrity and identifies injury types that may occur without visible bruising. However, such outcomes are not routinely integrated into standard carcass inspection systems, meaning that structural definitions of catch damage often appear in more specialized or experimental contexts.

In contrast, several large-scale analyses define catch damage indirectly through composite slaughter metrics, including carcass downgrades, condemnations, or DOA rates. In these cases, catch damage is embedded within broader indicators that also reflect transport stress, dehydration, thermal load, and underlying chicken pathology (Nijdam et al., 2006; van Emous et al., 2024; Whiting et al., 2007). Because these outcomes integrate multiple preslaughter processes, the resulting catch damage definition represents overall losses across the production chain, rather than a clearly defined injury outcome. Although DOA is frequently used as a practical welfare indicator within commercial monitoring systems, it represents a broad composite endpoint influenced not only by catching and handling, but also by environmental stressors, transport conditions, dehydration, metabolic and cardiovascular failure. Its specificity as an indicator of catch-related injury is therefore limited (Allen et al., 2023). A more specific iceberg indicator for catch-related damage may instead involve a composite slaughterhouse measure combining DOA with the prevalence of fresh traumatic lesions, particularly wing bruises and fractures identified as recent injuries.

Additional variation arises in studies that incorporate physiological indicators into the concept of catch damage. Measures such as corticosterone, glucose, lactate, CK, and body temperature primarily reflect physiological stress and systemic load rather than direct tissue injury. However, some markers, particularly CK activity, are commonly associated with skeletal muscle damage and may indirectly indicate an increased risk of handling-related injuries such as bruising or wing damage during catching and crating (Vošmerová et al., 2010; Yalçin et al., 2004). Similarly, studies examining transport microclimate have shown that severe environmental stress can increase mortality without a corresponding rise in bruising, demonstrating that physiological compromise and visible injury do not always occur at the same time (dos Santos et al., 2020). These parameters should therefore be regarded as proxy indicators. They are sensitive to preslaughter disturbance but do not specify whether, where, or when structural injuries developed. Their responses are influenced by many factors beyond catching mechanics, including temperature, dehydration, and chicken condition. Interpreting them as direct evidence of physical damage risks overestimating the traumatic component of handling. For meaningful interpretation, physiological results need to be considered alongside injury-based variables such as bruise size, color, anatomical location, and timing of observation. Physiological measures describe the condition of the animal, whereas lesions describe the structural outcome. The small number of studies combining both makes them particularly valuable, but also requires careful methodological investigation.

The reviewed literature demonstrates that catch damage has been investigated through several methodological pathways that differ not only technically but also conceptually in which dimensions of handling-related harm they capture. No single method provides a complete picture, and each involves trade-offs between temporal precision, biological specificity, feasibility, and scalability.

The most prevalent approach was postmortem injury scoring on the slaughter line, which offered considerable feasibility and scalability under commercial conditions. However, differences in lesion thresholds, color criteria, observer procedures, and sampling designs limited standardization between studies (Delanglez et al., 2025; Hassan and Lashin, 2017; Saraiva et al., 2020; Schilling et al., 2008). Injuries detected after plucking may also originate from several preslaughter stages rather than catching alone. Although lesion color may support approximate estimation of injury age, it cannot provide definitive stage attribution. Slaughter line scoring is therefore suitable for routine surveillance but less reliable for determining the precise origin of injuries.

Live chicken examinations, conducted immediately before or after loading provide an opportunity to assess injuries close to the catching event. By combining visual inspection with palpation and applying explicit injury size thresholds, these protocols aim to detect injuries that are most plausibly attributable to catching itself (Mönch et al., 2020; Wessel et al., 2022). Their major advantage lies in minimizing temporal confounding and in detecting structural injuries that may not manifest as obvious postmortem discolorations. However, these methods are labor intensive, sensitive to farm conditions, and difficult to implement on large commercial flocks (Meyer et al., 2020). They also risk underestimating injuries that become visible only after transport or processing. Thus, while this approach improves attribution of injury origin, it limits operational scalability.

Slaughterhouse records and certification schemes further increase operational applicability by using predefined defect thresholds and reporting prevalence at the load or flock level (Pirompud et al., 2023; van Emous et al., 2024). These systems offer repeatability and large datasets, however, aggregation rules (e.g., carcass level vs region-based counting) and scheme specific definitions substantially influence apparent prevalence. The resulting indicators are robust production metrics but biologically coarse, often unable to distinguish injury origin or severity.

In contrast, pathological and epidemiological frameworks capture severe trauma and mortality within broader diagnostic or risk modelling contexts (Caffrey et al., 2017; Kittelsen et al., 2015b). Their strength lies in identifying systemic pathology and modelling multifactorial risk, but they do not quantify injury size or frequency in a way that reflects sublethal injury burden. Mortality-based endpoints, in particular, are composite outcomes influenced by metabolic, cardiovascular, and environmental factors beyond handling.

The limited number of studies attempting to distinguish timing of catch damage occurrence highlights one of the most critical methodological gaps in catch damage research. Although several practical strategies exist, most rely on macroscopic interpretation rather than quantitative aging techniques, and therefore operate within biological uncertainty. Observer-based color and morphology classification, as applied in slaughterhouse-monitoring frameworks, offers a practical and scalable solution. By combining bruise color, injury appearance, and anatomical distribution with knowledge of stage-specific injury patterns, assessors can approximate whether damage is likely to have occurred during rearing, catching, transport, or processing (Cockram et al., 2020). This approach is practical but depends on observer judgement and gradual color changes. Even early experimental studies showed that bruise colors overlap over time and are affected by factors such as tissue depth, location, and processing, meaning that visual aging can only give an estimate rather than an exact result (Hamdy et al., 1961a,; 1961b; Northcutt et al., 2000).

Restrictive criteria based on lesion size, red coloration, and visible hemorrhage may improve the consistency and specificity of recent injury classification (Delanglez et al., 2025). However, these criteria remain dependent on visible surface characteristics and may exclude injuries that are less externally evident or have already begun to resolve. Assessments conducted shortly after catching and compared with baseline observations collected before catching can reduce temporal uncertainty (Wessel et al., 2022), but they are difficult to implement on a commercial scale. Moreover, some injuries may be obscured by feathers and only become clearly visible after plucking, which complicates direct comparison between live-bird assessments and postmortem estimates. Conversely, assessments performed only after plucking may identify lesions more clearly but provide less certainty regarding the stage at which the injury occurred. Combining observations collected at successive production stages with objective measurements and biological validation may therefore provide a more reliable basis for injury attribution.

From an industry perspective, the ability to determine when damage occurred is of high practical importance. Stage specific attribution has implications for contractual responsibility, welfare auditing, and targeted intervention. Distinguishing whether injuries originated on-farm, during loading, in transport, or within the slaughterhouse affects both management decisions and economic accountability. Current methods provide partial but not definitive answers to this question, limiting their utility in resolving disputes or guiding precise corrective action. Emerging technologies could address some of these limitations through continuous and standardized monitoring. Machine-learning approaches based on flock-level and preslaughter-level variables may further support risk-based prediction of bruising prevalence, although they do not directly detect or attribute individual injuries (Pirompud et al., 2025). Vision systems enabled by the Internet of Things may combine real-time image acquisition with dynamic tracking of broilers or transport modules across successive production stages (Lin and Suhendra, 2025). Automated image analysis could quantify lesion size, color, anatomical location, hemorrhage characteristics, and abnormal wing position while reducing observer subjectivity (Langkabel et al., 2015; Libera et al., 2025; Lyu et al., 2025; van Staaveren et al., 2026). Linking image data with sensor information on impacts, movement, temperature, humidity, transport duration, and processing conditions could further identify events associated with increased injury risk. Tracking the same broilers or transport modules before and after loading, arrival, shackling, and plucking could also help establish when an injury first becomes detectable. However, automated systems remain sensitive to imaging conditions and are generally limited to externally visible injuries (Chowdhury and Morey, 2020). Reliable stage specific attribution would therefore require standardized image acquisition, synchronized data collection, stable tracking, and external validation across commercial settings. Algorithm performance should be reported using measures such as sensitivity, specificity, repeatability, and agreement with trained observers. Automated classifications should also be validated against controlled bruise aging models, histological findings, or confirmed fracture diagnoses (Northcutt et al., 2000; Voogt et al., 2023). Combining continuous automated monitoring with targeted biological validation may therefore offer the most realistic pathway toward objective and stage specific injury assessment.

A standardized catch damage assessment protocol should clearly specify the injury definitions, assessment stage, anatomical regions, lesion thresholds, counting unit, sampling procedure, and observer training. Bruises, fractures, dislocations, and DOA should be reported separately because they represent different outcomes and may originate at different production stages. Whenever feasible, assessments should be conducted at more than one stage, and agreement between observers should be evaluated. These elements would improve comparability between studies and strengthen the attribution of injuries to specific handling procedures.

When considering the evidence as a whole, the methodological strength of the included studies varied considerably. Controlled experimental studies generally provided better control of potential confounding factors but often involved smaller samples, whereas commercial field and slaughterhouse studies included larger populations but were more susceptible to confounding and frequently relied on aggregated data. Differences in injury definitions, diagnostic thresholds, assessment stages, sampling procedures, and reporting units further reduced comparability between studies. Consequently, the conclusions should be interpreted as a synthesis of recurring methodological patterns rather than a quantitative ranking of the reliability of individual assessment methods.

Taken together, the existing literature underscores the importance of catch damage as a key welfare and quality indicator, while highlighting persistent methodological limitations that constrain both scientific comparability and practical application. In particular, the lack of standardized assessment protocols and limited ability to determine the stage of injury occurrence remain critical barriers. Addressing these challenges through harmonized definitions, standardized assessment frameworks, and improved methods for stage-specific attribution will be essential to strengthen the reliability and practical value of catch damage as an indicator in both research and commercial practice.

## Conclusions

This review demonstrates that catch damage in broiler chickens should be understood not only as a biological outcome of physical injury, but also as a measurement construct shaped by methodological choices, assessment stage, injury definitions, scoring criteria, and attribution procedures. Across the literature, it encompasses diverse endpoints, including bruising, fractures, dislocations, carcass condemnations, and mortality indicators, which differ in pathogenesis, stage of injury occurrence, and detection sensitivity. A key limitation is the reliance on postmortem proxy measures, which reflect cumulative trauma across multiple preslaughter stages rather than catching-specific injury. Limited ability to differentiate between fresh and old lesions further restricts stage-specific attribution and weakens causal inference. In addition, heterogeneity in injury definitions, lesion thresholds, anatomical coverage, scoring systems, and reporting practices introduces measurement bias and limits reproducibility and cross-study comparability. Overall, methodological design strongly determines reported catch damage outcomes, and no single approach provides a complete or stage-specific assessment. Progress in this field requires harmonized definitions, standardized scoring protocols, and validated methods for injury age determination. Such advances are essential to improve the reliability, comparability, and practical applicability of catch damage as an indicator of broiler welfare and carcass quality.

## Author contributions

**P. Sztandarski** contributed to the conception and design of the study, development of the methodology, investigation, data curation and formal analysis, visualization of the results, and preparation of the original manuscript draft. **J. Marchewka** contributed to the conception and design of the study, development of the methodology, supervision and project administration, acquisition of funding, and critical review and revision of the manuscript for important intellectual content. **A. Jaszczyk** contributed to the conception and design of the study, development of the methodology, investigation, data curation and formal analysis, visualization of the results, and preparation of the original manuscript draft. **M. Solka** contributed to the methodology, investigation, data curation and validation of the work, and critically reviewed and revised the manuscript for important intellectual content. **J.O. Horbańczuk** contributed to the methodology, investigation, data curation and validation of the work, and critically reviewed and revised the manuscript for important intellectual content. **A.B. Riber** contributed to the methodology, investigation, data curation and validation of the work, and critically reviewed and revised the manuscript for important intellectual content. **F.A.M. Tuyttens** contributed to the conception and design of the study, development of the methodology, supervision and acquisition of funding, and critically reviewed and revised the manuscript for important intellectual content. **T.B. Rodenburg** contributed to the conception and design of the study, development of the methodology, supervision and project administration, acquisition of funding, and critically reviewed and revised the manuscript for important intellectual content.

All authors reviewed and explicitly approved the final version of the manuscript and agree to be personally and publicly accountable for all aspects of the work, including ensuring that any questions regarding the accuracy or integrity of the work are appropriately investigated and resolved.

## Ethical statement

Not applicable.

## Disclosures

The authors declare no competing interests.

## References

[bib0001] Allen S.E., Parker C.D., Verheyen K.L.P., Nicol C.J., Chang Y.M. (2023). Effects of external ambient temperature at loading, journey duration and flock characteristics on the dead-on-arrival rate in broiler chickens transported to slaughter in Great Britain. Poult. Sci..

[bib0002] Averós X., Balderas B., Cameno E., Estevez I. (2020). The value of a retrospective analysis of slaughter records for the welfare of broiler chickens. Poult. Sci..

[bib0003] Buzdugan S.N., Chang Y.M., Huntington B., Rushton J., Guitian J., Alarcon P., Blake D.P. (2020). Identification of production chain risk factors for slaughterhouse condemnation of broiler chickens. Prev. Vet. Med..

[bib0004] Caffrey N.P., Dohoo I.R., Cockram M.S. (2017). Factors affecting mortality risk during transportation of broiler chickens for slaughter in Atlantic Canada. Prev. Vet. Med..

[bib0005] Chauvin C., Hillion S., Balaine L., Michel V., Peraste J., Petetin I. (2011). Factors associated with mortality of broilers during transport to slaughterhouse. Animal.

[bib0006] Chowdhury E.U., Morey A. (2020). Application of optical technologies in the US poultry slaughter facilities for the detection of poultry carcase condemnation. Br. Poult. Sci..

[bib0007] Cockram M.S., Dulal K.J., Mohamed R.A., Revie C.W. (2018). Risk factors for bruising and mortality of broilers during manual handling, module loading, transport, and lairage. Can. J. Anim. Sci..

[bib0008] Cockram M.S., Dulal K.J., Stryhn H., Revie C.W. (2020). Rearing and handling injuries in broiler chickens and risk factors for wing injuries during loading. Can. J. Anim. Sci..

[bib0009] De Lima V.A., Ceballos M.C., Gregory N.G., Da Costa M.J.P. (2019). Effect of different catching practices during manual upright handling on broiler welfare and behavior. Poult. Sci..

[bib0010] Delanglez F., Watteyn A., Ampe B., Segers V., Garmyn A., Delezie E. (2025). Comparing methods for catching and crating broiler chicken flocks: a trade-off between animal welfare, ergonomics and economics. Poult. Sci..

[bib0011] Dos Santos V.M., Dallago B.S., Racanicci A.M., Santana Â.P., Cue R.I., Bernal F.E. (2020). Effect of transportation distances, seasons and crate microclimate on broiler chicken production losses. PLoS One.

[bib0012] Gou Z., Abouelezz K.F.M., Fan Q., Li L., Lin X., Wang Y. (2021). Physiological effects of transport duration on stress biomarkers and meat quality of medium-growing Yellow broiler chickens. Animal.

[bib0013] Gouveia K.G., Vaz-Pires P., da Costa P.M. (2009). Welfare assessment of broilers through examination of haematomas, foot-pad dermatitis, scratches and breast blisters at processing. Anim. Welf..

[bib0015] Hamdy M.K., May K.N., Flanagan W.P., Powers J.J. (1961). Determination of the age of bruises in chicken broilers. Poult. Sci..

[bib0016] Hamdy M.K., May K.N., Powers J.J. (1961). Some physical and physiological factors affecting poultry bruises. Poult. Sci..

[bib0017] Hassan F.A., Lashin A.I. (2017). Influence of handling processes for broilers on mortality and carcass quality at slaughterhouse. Misr J. Agric. Eng..

[bib0018] IKB Kip (2017). Bijlage 8 beoordelingssysteem vleeskuikens. https://www.avined.nl/wp-content/uploads/8-Beoordelingssysteem-vleeskuikens-IKB-Kip-versie-6-210601.pdf.

[bib0014] Jacobs L., Tuyttens F.A.M., Nicol C. (2020).

[bib0019] Jacobs L., Delezie E., Duchateau L., Goethals K., Tuyttens F.A. (2017). Impact of the separate pre-slaughter stages on broiler chicken welfare. Poult. Sci..

[bib0020] Kittelsen K.E., Granquist E.G., Aunsmo A.L., Moe R.O., Tolo E. (2018). An evaluation of two different broiler catching methods. Animals.

[bib0021] Kittelsen K.E., Granquist E.G., Kolbjørnsen Ø., Nafstad O., Moe R.O. (2015). A comparison of post-mortem findings in broilers dead-on-farm and broilers dead-on-arrival at the abattoir. Poult. Sci..

[bib0022] Kittelsen K.E., Granquist E.G., Vasdal G., Tolo E., Moe R.O. (2015). Effects of catching and transportation versus pre-slaughter handling at the abattoir on the prevalence of wing fractures in broilers. Anim. Welf..

[bib0023] Lambrecht E., Jacobs L., Delezie E., De Steur H., Gellynck X., Tuyttens F.A.M. (2020). Stakeholder perceptions on broiler chicken welfare during first-day processing and the pre-slaughter phase: a case study in Belgium. World's. Poult. Sci. J..

[bib0024] Langkabel N., Baumann M.P., Feiler A., Sanguankiat A., Fries R. (2015). Influence of two catching methods on the occurrence of lesions in broilers. Poult. Sci..

[bib0025] Libera K., Schut D., Kritsi E., van Steijn L., Dallman T., Lipman L. (2025). Detecting wing fractures in chickens using deep learning, photographs and computed tomography scanning. Poult. Sci..

[bib0026] Lin H.T., Suhendra (2025). Development and implementation of an IoT-enabled smart poultry slaughtering system using dynamic object tracking and recognition. Sensors.

[bib0027] Lyu Y., Wu F., Wang Q., Liu G., Zhang Y., Jiang H., Zhou M. (2025). A review of robotic and automated systems in meat processing. Front. Robot. AI.

[bib0028] Malpass M.C., Williams A.P., Jones D.L., Omed H.M. (2010). Microbiological quality of chicken wings damaged on the farm or in the processing plant. Food Microbiol..

[bib0029] Meyer M.M., Johnson A.K., Bobeck E.A. (2020). Development and validation of broiler welfare assessment methods for research and on-farm audits. J. Appl. Anim. Welf. Sci..

[bib0030] Mönch J., Rauch E., Hartmannsgruber S., Erhard M., Wolff I., Schmidt P. (2020). The welfare impacts of mechanical and manual broiler catching and of circumstances at loading under field conditions. Poult. Sci..

[bib0031] Nielsen S.S., Alvarez J., Bicout D.J., Calistri P., Canali E., EFSA AHAW Panel (EFSA Panel on Animal Health and Welfare) (2023). Welfare of broilers on farm. EFSA J.

[bib0032] Nijdam E., Delezie E., Lambooij E., Nabuurs M.J., Decuypere E., Stegeman J.A. (2005). Comparison of bruises and mortality, stress parameters, and meat quality in manually and mechanically caught broilers. Poult. Sci..

[bib0033] Nijdam E., Zailan A.R., Van Eck J.H., Decuypere E., Stegeman J.A. (2006). Pathological features in dead on arrival broilers with special reference to heart disorders. Poult. Sci..

[bib0034] Northcutt J.K., Buhr R.J., Rowland G.N. (2000). Relationship of broiler bruise age to appearance and tissue histological characteristics. J. Appl. Poult. Res..

[bib0035] Page M.J., McKenzie J.E., Bossuyt P.M., Boutron I., Hoffmann T.C., Mulrow C.D. (2021). The PRISMA 2020 statement: an updated guideline for reporting systematic reviews. BMJ.

[bib0036] Petracci M., Bianchi M., Cavani C. (2010). Pre-slaughter handling and slaughtering factors influencing poultry product quality. World's. Poult. Sci. J..

[bib0037] Pilecco M., Almeida Paz I.C.L., Tabaldi L.A., Nääs I.A., Garcia R.G., Caldara F.R., Francisco N.S. (2013). Training of catching teams and reduction of back scratches in broilers. Braz. J. Poult. Sci..

[bib0038] Pirompud P., Sivapirunthep P., Punyapornwithaya V., Chaosap C. (2023). Preslaughter handling factors affecting dead on arrival, condemnations, and bruising in broiler chickens raised without an antibiotic program. Poult. Sci..

[bib0039] Pirompud P., Sivapirunthep P., Punyapornwithaya V., Chaosap C. (2025). Predictive modeling of bruising in broiler chickens using machine learning algorithms. Poult. Sci..

[bib0040] Pitcovski J., Ohana N., Amzaleg L., Krispel S., Bercovich D., Pinchasov Y. (1997). Identification of a starting point of breast skin tears during chicken plucking. Poult. Sci..

[bib0041] PVE. 2001. Beoordelingssysteem Kwaliteit Vleeskuikens. Product Boards for Livestock Meat and Eggs, Zoetermeer, The Netherlands.

[bib0042] Saraiva S., Esteves A., Oliveira I., Mitchell M., Stilwell G. (2020). Impact of pre-slaughter factors on welfare of broilers. Vet. Anim. Sci..

[bib0043] Schilling M.W., Radhakrishnan V., Thaxton Y.V., Christensen K., Thaxton J.P., Jackson V. (2008). The effects of broiler catching method on breast meat quality. Meat. Sci..

[bib0044] Smith K.C., Pierdon M.K. (2025). Impact of mechanical vs. manual catching on stress, fear, and carcass quality in slower growing broilers. J. Appl. Anim. Welf. Sci..

[bib0045] van Emous R.A., van Harn J., van Riel J.W. (2024). Effects of season, catching method, and thinning on carcass quality and production parameters in 4 different broiler production systems in the Netherlands. Poult. Sci..

[bib0046] van Staaveren N., van Langeveld K., Schulte-Landwehr J., Mulder J., Galliot P., Créach P., Sztandarski P., Marchewka J., Forkman B., Van Noten N., Tuyttens F.A.M., Giersberg M.F., Manet M.W.E., Rodenburg T.B. (2026). Validation of an automated system for at-slaughter assessment of footpad dermatitis and hock burn in broiler chickens. Poult. Sci..

[bib0047] Voogt A.M., Schrijver R.S., Temürhan M., Bongers J.H., Sijm D.T. (2023). Opportunities for regulatory authorities to assess animal-based measures at the slaughterhouse using sensor technology and artificial intelligence: a review. Animals.

[bib0048] Vošmerová P., Bedáňová I., Chloupek P., Chloupek J., Suchý P., Večerek V. (2010). Transport-induced changes in selected biochemical indices in broilers as affected by ambient temperatures. Acta Vet. Brno.

[bib0049] Welfare Quality (2009).

[bib0050] Wessel J., Rauch E., Hartmannsgruber S., Erhard M., Schmidt P., Schade B., Louton H. (2022). A comparison of two manual catching methods of broiler considering injuries and behavior. Poult. Sci..

[bib0051] Whiting T.L., Drain M.E., Rasali D.P. (2007). Warm weather transport of broiler chickens in Manitoba. II. Truck management factors associated with death loss in transit to slaughter. Can. Vet. J..

[bib0052] Wilcox C.H., Sandilands V., Mayasari N., Asmara I.Y., Anang A. (2024). A literature review of broiler chicken welfare, husbandry, and assessment. World's. Poult. Sci. J..

[bib0053] Yalçin S., Özkan S., Oktay G., Çabuk M., Erbayraktar Z., Bilgili S.F. (2004). Age-related effects of catching, crating, and transportation at different seasons on core body temperature and physiological blood parameters in broilers. J. Appl. Poult. Res..

